# *catena*-Poly[[aqua­sodium(I)]-μ-[2,2′-(disulfanedi­yl)bis­(pyridine *N*-oxide)]-μ-(pyridine-2-thiol­ato 1-oxide)]

**DOI:** 10.1107/S1600536810000073

**Published:** 2010-01-13

**Authors:** B. Ravindran Durai Nayagam, Samuel Robinson Jebas, J. Jebaraj Devadasan, R. Murugesan, Dieter Schollmeyer

**Affiliations:** a Department of Chemistry, Popes College, Sawyerpuram 628 251, Tamilnadu, India; b Department of Physics, Sethupathy Government Arts College, Ramanathapuram 623 502, Tamilnadu, India; c Department of Physics, Popes College, Sawyerpuram 628 251, Tamilnadu, India; d Department of Chemistry, T.D.M.N.S. College, T. Kallikulam, Tamilnadu, India; e Institut für Organische Chemie, Universität Mainz, Duesbergweg 10-14, 55099 Mainz, Germany

## Abstract

There are two monomeric units in the asymmetric unit of the polymeric title compound, [Na(C_5_H_4_NOS)(C_10_H_8_N_2_O_2_S_2_)(H_2_O)]_*n*_. The Na^I^ ions are six coordinated by four O atoms, one S atom and one water mol­ecule, forming a slightly distorted octa­hedral geometry. An intra­molecular O—H⋯O hydrogen bond stabilizes the conformation of the mol­ecule. The crystal packing is consolidated by inter­molecular O—H⋯O, O—H⋯N and O—H⋯S hydrogen bonds, π–π inter­actions [with centroid–centroid distances of 3.587 (2) Å] together with weak C—H⋯π inter­actions. The mol­ecules are linked into polymeric chains along the *b*-axis direction.

## Related literature

For the biological activity of *N*-oxides and their derivatives, see: Lobana & Bhatia (1989 ▶); Symons *et al.* (1985 ▶). For their involvement in DNA strand scission under physiological conditions, see: Katsuyuki *et al.* (1991 ▶); Bovin *et al.* (1992 ▶). Pyridine *N*-oxides bearing a sulfur group in position two display significant anti­microbial activity, see: Leonard *et al.* (1955 ▶). For related structures, see: Jebas *et al.* (2005 ▶); Ravindran *et al.* (2008 ▶).
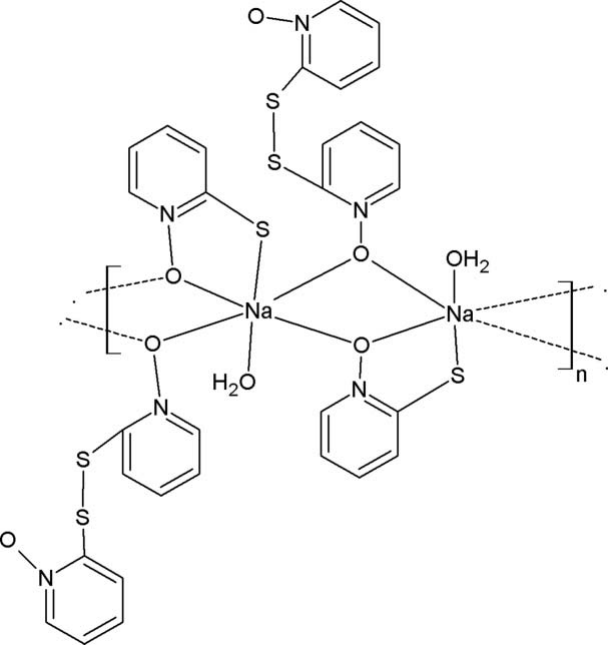


## Experimental

### 

#### Crystal data

[Na(C_5_H_4_NOS)(C_10_H_8_N_2_O_2_S_2_)(H_2_O)]
*M* *_r_* = 838.92
Orthorhombic, 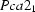

*a* = 24.829 (2) Å
*b* = 7.3290 (7) Å
*c* = 19.1378 (17) Å
*V* = 3482.5 (5) Å^3^
*Z* = 4
Mo *K*α radiation
μ = 0.48 mm^−1^
*T* = 173 K
0.54 × 0.19 × 0.14 mm

#### Data collection

Bruker SMART APEXII CCD diffractometer
Absorption correction: multi-scan (*SADABS*; Sheldrick, 2008*a* ▶) *T*_min_ = 0.782, *T*_max_ = 0.936
38861 measured reflections
8403 independent reflections
6205 reflections with *I* > 2σ(*I*)
*R*_int_ = 0.077

#### Refinement

*R*[*F* ^2^ > 2σ(*F* ^2^)] = 0.037
*wR*(*F* ^2^) = 0.074
*S* = 0.93
8403 reflections
469 parameters
1 restraint
H-atom parameters constrained
Δρ_max_ = 0.28 e Å^−3^
Δρ_min_ = −0.23 e Å^−3^
Absolute structure: Flack (1983 ▶), 4068 Friedel pairs
Flack parameter: 0.47 (6)

### 

Data collection: *APEX2* (Bruker, 2008 ▶); cell refinement: *SAINT* (Bruker, 2008 ▶); data reduction: *SAINT*; program(s) used to solve structure: *SHELXS97* (Sheldrick, 2008*b* ▶); program(s) used to refine structure: *SHELXL97* (Sheldrick, 2008*b* ▶); molecular graphics: *SHELXTL* (Sheldrick, 2008*b* ▶); software used to prepare material for publication: *SHELXTL* and *PLATON* (Spek, 2009 ▶).

## Supplementary Material

Crystal structure: contains datablocks global, I. DOI: 10.1107/S1600536810000073/bt5158sup1.cif

Structure factors: contains datablocks I. DOI: 10.1107/S1600536810000073/bt5158Isup2.hkl

Additional supplementary materials:  crystallographic information; 3D view; checkCIF report

## Figures and Tables

**Table 1:** Hydrogen-bond geometry (Å, °) *Cg*1 is the centroid of the N34/C33–C35 ring.

*D*—H⋯*A*	*D*—H	H⋯*A*	*D*⋯*A*	*D*—H⋯*A*
O49—H49*A*⋯O16^i^	0.90	1.91	2.784 (3)	165
O49—H49*A*⋯N11^i^	0.90	2.67	3.417 (3)	141
O49—H49*B*⋯S32^ii^	0.89	2.37	3.198 (2)	154
O50—H50*A*⋯O39^iii^	0.92	1.88	2.798 (3)	177
O50—H50*A*⋯N34^iii^	0.92	2.62	3.455 (3)	151
O50—H50*B*⋯S24	0.86	2.33	3.193 (2)	174
C29—H29⋯*Cg*1^iv^	0.95	2.89	3.618 (4)	134

Symmetry codes: (i) 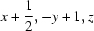; (ii) 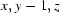; (iii) 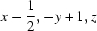; (iv) 
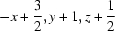
.

**Fig. 1.**
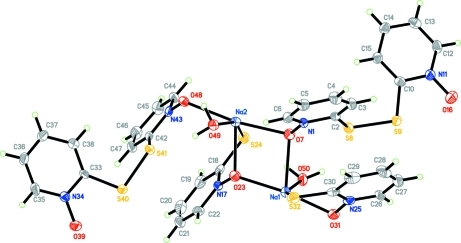
The molecular structure of the title compound, showing 50% probability displacement ellipsoids and the atom numbering scheme.

**Fig. 2.**
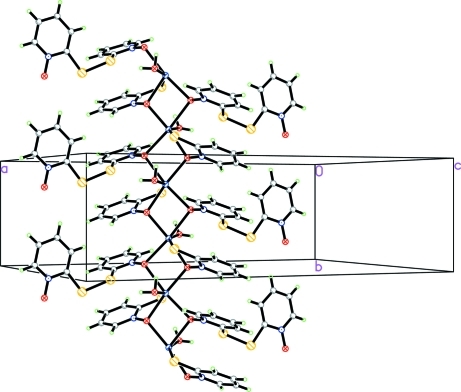
The crystal packing of the title compound, showing polymeric chains along the b-direction.

## supplementary crystallographic information

### Comment

*N*-oxides and their derivatives show a broad spectrum of biological activity such as antifungal, antimicrobial and antibacterial activities (Lobana & Bhatia, 1989; Symons *et al.*, 1985). These compounds are also found to be involved in DNA strand scission under physiological conditions (Katsuyuki *et al.*, 1991; Bovin *et al.*, 1992). Pyridine *N*-oxides bearing a sulfur group in position two display significant antimicrobial activity (Leonard *et al.*, 1955).  In view of the importance of *N*-oxides, we have previously reported the crystal structures of *N*-oxide derivatives (Jebas *et al.*, 2005; Ravindran *et al.*, 2008). As an extension of our work on *N*-oxide derivatives, we report here the crystal structure of the title compound (I).

In the asymmetric unit of (I)(Fig 1), the Na^I^ ion is six coordinated by four oxygen atoms, two from 1-oxypyridine-2-thiolato and two from 2,2-thiobis(pyridine *N*-oxide) ligands, and one sulfur atom from 1-oxypyridine-2-thiolato ligand. The pyridyl rings are essentially planar with the maximum deviation from planarity of -0.034 (5) Å for atom C10. The N–O bond lengths are in good agreement with the mean value of 1.304 (15) Å reported in the literature for pyridine *N*-oxides.

Intramolecular O—H···O hydrogen bonding influence the conformation of the molecule. The crystal packing (Fig 2) is consolidated by intermolecular O—H···O, O—H···N and O—H···S hydrogen bonding together with intramolecular S···O= 2.587 (3) to 3.069 (3) Å; N···S=2.686 (4) to 2.700 (5) Å; S···Na=3.807 (2) Å, intermolecular S···O^i^=3.065 (3) Å, O···O^i^=2.784 (3), O···O^ii^=2.798 (3) Å, intramolecular S···O =2.587 (3)to 3.069 (3) Å; O···O = 2.784 (3) to 2.798 (3) Å; S···Na=3.807 (2) Å and N···S=2.686 (4) to 2.700 (5) Å short contacts [symmetry code: (i) 1/2+*X*,1-Y,*Z* (ii) -1/2+*X*, 1-Y, *Z*]. π—π interactions with cg1-cg4^iii^=3.587 (2)Å (Where *Cg*1 is N1/C2—C6; *Cg*2 is N25/C26—C30) [symmetry code: (iii) *X*, -1+Y, *Z*] together with weak C—H··· π interactions. The molecules are linked into polymeric chains along the b-direction.

### Experimental

A  mixture of Sodium salt of 1-hydroxypyidine-2-thione  (0.298 2mmol), ethanol (10 ml)and sodium ethoxide (10ml) was heated at 333 K with stirring for 30 min. After two days again Sodium salt of 1-hydroxypyidine-2-thione(0.149,1mmol)was added. The solution was again  heated at 333 K with stirring for 30 min. The mixture was then kept aside for slow evaporation. After a week colourless crystals were formed.

### Refinement

After checking their presence in the Fourier map, all the aromatic hydrogen atoms were fixed on the calculated positions and allowed to ride on their parent atoms with the C—H = 0.95 Å. The water hydrogen atoms were located from the Fourier map and allowed to refine freely with the distances O—H = 0.86 to 0.92Å (water) with *U*_iso_(C) in the range of 1.2U_equ_(C) and 1.5U_equ_(O) water. The crystal was an inversion twin. The Flack parameter indicates the fractional contribution of the twin components.

### Crystal data

**Table:**

[Na(C_5_H_4_NOS)(C_10_H_8_N_2_O_2_S_2_)(H_2_O)]	*F*(000) = 1728
*M**_r_* = 838.92	*D*_x_ = 1.600 Mg m^−^^3^
Orthorhombic, *P**c**a*2_1_	Mo *K*α radiation, λ = 0.71069 Å
Hall symbol: P 2c -2ac	Cell parameters from 6853 reflections
*a* = 24.829 (2) Å	θ = 2.7–27.9°
*b* = 7.3290 (7) Å	µ = 0.48 mm^−^^1^
*c* = 19.1378 (17) Å	*T* = 173 K
*V* = 3482.5 (5) Å^3^	Block, colorless
*Z* = 4	0.54 × 0.19 × 0.14 mm

### Data collection

**Table:**

Bruker SMART APEXII CCD diffractometer	8403 independent reflections
Radiation source: sealed Tube	6205 reflections with *I* > 2σ(*I*)
graphite	*R*_int_ = 0.077
CCD scan	θ_max_ = 28.0°, θ_min_ = 1.6°
Absorption correction: multi-scan (*SADABS*; Sheldrick, 2008a)	*h* = −32→30
*T*_min_ = 0.782, *T*_max_ = 0.936	*k* = −9→9
38861 measured reflections	*l* = −25→25

### Refinement

**Table:**

Refinement on *F*^2^	Secondary atom site location: difference Fourier map
Least-squares matrix: full	Hydrogen site location: calc
*R*[*F*^2^ > 2σ(*F*^2^)] = 0.037	H-atom parameters constrained
*wR*(*F*^2^) = 0.074	*w* = 1/[σ^2^(*F*_o_^2^) + (0.0277*P*)^2^] where *P* = (*F*_o_^2^ + 2*F*_c_^2^)/3
*S* = 0.93	(Δ/σ)_max_ < 0.001
8403 reflections	Δρ_max_ = 0.28 e Å^−^^3^
469 parameters	Δρ_min_ = −0.23 e Å^−^^3^
1 restraint	Absolute structure: Flack (1983), 4068 Friedel pairs
Primary atom site location: structure-invariant direct methods	Flack parameter: 0.47 (6)

### Special details

**Table:**

Geometry. All e.s.d.'s (except the e.s.d. in the dihedral angle between two l.s. planes) are estimated using the full covariance matrix. The cell e.s.d.'s are taken into account individually in the estimation of e.s.d.'s in distances, angles and torsion angles; correlations between e.s.d.'s in cell parameters are only used when they are defined by crystal symmetry. An approximate (isotropic) treatment of cell e.s.d.'s is used for estimating e.s.d.'s involving l.s. planes.
Refinement. Refinement of *F*^2^ against ALL reflections. The weighted *R*-factor *wR* and goodness of fit *S* are based on *F*^2^, conventional *R*-factors *R* are based on *F*, with *F* set to zero for negative *F*^2^. The threshold expression of *F*^2^ > σ(*F*^2^) is used only for calculating *R*-factors(gt) *etc*. and is not relevant to the choice of reflections for refinement. *R*-factors based on *F*^2^ are statistically about twice as large as those based on *F*, and *R*- factors based on ALL data will be even larger.

### Fractional atomic coordinates and isotropic or equivalent isotropic displacement parameters (Å^2^)

**Table:**

	*x*	*y*	*z*	*U*_iso_*/*U*_eq_	
Na1	0.62174 (8)	0.7233 (3)	0.43117 (11)	0.0209 (4)	
Na2	0.63035 (8)	0.2253 (3)	0.42791 (11)	0.0205 (4)	
N1	0.55812 (13)	0.4551 (4)	0.55069 (17)	0.0169 (6)	
C2	0.5074 (3)	0.5199 (5)	0.5651 (4)	0.0192 (12)	
C3	0.4871 (3)	0.5177 (5)	0.6318 (4)	0.0254 (14)	
H3	0.4518	0.5612	0.6413	0.031*	
C4	0.51902 (17)	0.4511 (5)	0.6844 (2)	0.0284 (8)	
H4	0.5059	0.4482	0.7310	0.034*	
C5	0.57028 (13)	0.3880 (5)	0.66984 (17)	0.0261 (8)	
H5	0.5923	0.3419	0.7064	0.031*	
C6	0.58936 (13)	0.3919 (4)	0.60254 (17)	0.0233 (7)	
H6	0.6247	0.3498	0.5927	0.028*	
O7	0.57443 (13)	0.4583 (4)	0.48528 (17)	0.0221 (6)	
S8	0.47870 (3)	0.60118 (11)	0.48539 (5)	0.02044 (17)	
S9	0.40763 (3)	0.71334 (11)	0.51954 (5)	0.02278 (18)	
C10	0.3632 (2)	0.5278 (6)	0.5114 (3)	0.0194 (10)	
N11	0.31067 (11)	0.5830 (4)	0.50875 (14)	0.0217 (6)	
C12	0.27083 (19)	0.4613 (6)	0.4983 (3)	0.0257 (10)	
H12	0.2348	0.5032	0.4931	0.031*	
C13	0.28145 (15)	0.2766 (6)	0.49496 (19)	0.0247 (9)	
H13	0.2530	0.1922	0.4873	0.030*	
C14	0.33365 (14)	0.2158 (5)	0.50285 (17)	0.0245 (8)	
H14	0.3414	0.0889	0.5026	0.029*	
C15	0.37491 (14)	0.3433 (5)	0.51114 (17)	0.0208 (7)	
H15	0.4111	0.3034	0.5166	0.025*	
O16	0.30158 (11)	0.7595 (4)	0.51612 (17)	0.0334 (7)	
N17	0.68938 (18)	0.4697 (4)	0.3143 (2)	0.0169 (8)	
C18	0.65984 (13)	0.3867 (4)	0.26226 (16)	0.0202 (7)	
C19	0.68407 (14)	0.3771 (5)	0.19601 (17)	0.0263 (8)	
H19	0.6645	0.3237	0.1585	0.032*	
C20	0.73511 (18)	0.4424 (6)	0.1836 (2)	0.0320 (9)	
H20	0.7508	0.4317	0.1385	0.038*	
C21	0.7630 (2)	0.5230 (5)	0.2370 (3)	0.0292 (13)	
H21	0.7981	0.5705	0.2288	0.035*	
C22	0.7405 (2)	0.5353 (5)	0.3019 (3)	0.0234 (10)	
H22	0.7603	0.5895	0.3390	0.028*	
O23	0.67089 (18)	0.4834 (3)	0.3798 (3)	0.0219 (9)	
S24	0.59697 (3)	0.30128 (11)	0.28014 (5)	0.02486 (19)	
N25	0.5601 (2)	0.9741 (4)	0.5395 (3)	0.0210 (9)	
C26	0.51021 (18)	1.0416 (6)	0.5488 (2)	0.0223 (9)	
H26	0.4923	1.0962	0.5103	0.027*	
C27	0.4846 (2)	1.0345 (6)	0.6115 (3)	0.0295 (11)	
H27	0.4490	1.0807	0.6166	0.035*	
C28	0.5116 (2)	0.9575 (6)	0.6683 (2)	0.0347 (10)	
H28	0.4948	0.9502	0.7128	0.042*	
C29	0.56242 (15)	0.8933 (5)	0.65881 (19)	0.0320 (8)	
H29	0.5810	0.8448	0.6980	0.038*	
C30	0.58885 (13)	0.8950 (4)	0.59367 (17)	0.0230 (7)	
O31	0.58128 (18)	0.9846 (3)	0.4763 (2)	0.0212 (9)	
S32	0.65125 (3)	0.80270 (12)	0.57957 (5)	0.0296 (2)	
C33	0.8877 (2)	0.0209 (5)	0.3402 (3)	0.0169 (10)	
N34	0.93985 (10)	0.0766 (4)	0.34558 (13)	0.0190 (6)	
C35	0.97985 (18)	−0.0463 (6)	0.3550 (2)	0.0258 (9)	
H35	1.0159	−0.0058	0.3612	0.031*	
C36	0.96829 (16)	−0.2304 (6)	0.3558 (2)	0.0292 (10)	
H36	0.9966	−0.3161	0.3620	0.035*	
C37	0.91595 (14)	−0.2915 (5)	0.34758 (18)	0.0269 (8)	
H37	0.9080	−0.4183	0.3469	0.032*	
C38	0.87541 (14)	−0.1622 (5)	0.34031 (18)	0.0233 (7)	
H38	0.8390	−0.2004	0.3354	0.028*	
O39	0.94960 (10)	0.2526 (4)	0.33994 (15)	0.0264 (6)	
S40	0.84312 (3)	0.20813 (11)	0.33478 (5)	0.02221 (17)	
S41	0.77227 (3)	0.09806 (11)	0.37045 (5)	0.01945 (17)	
C42	0.7423 (3)	0.0149 (4)	0.2941 (4)	0.0157 (12)	
N43	0.69222 (13)	−0.0467 (4)	0.30792 (17)	0.0180 (7)	
C44	0.65899 (13)	−0.1106 (4)	0.25716 (17)	0.0219 (7)	
H44	0.6236	−0.1497	0.2684	0.026*	
C45	0.67693 (13)	−0.1180 (5)	0.18980 (18)	0.0271 (8)	
H45	0.6539	−0.1613	0.1538	0.033*	
C46	0.72926 (16)	−0.0620 (5)	0.17386 (19)	0.0265 (8)	
H46	0.7423	−0.0700	0.1273	0.032*	
C47	0.7618 (3)	0.0052 (4)	0.2263 (4)	0.0214 (14)	
H47	0.7974	0.0445	0.2161	0.026*	
O48	0.67633 (12)	−0.0393 (3)	0.37434 (17)	0.0203 (6)	
O49	0.69056 (8)	0.1936 (3)	0.52554 (13)	0.0271 (5)	
H49A	0.7262	0.1920	0.5174	0.041*	
H49B	0.6880	0.0728	0.5310	0.041*	
O50	0.56110 (8)	0.6934 (3)	0.33598 (13)	0.0257 (5)	
H50A	0.5244	0.7115	0.3356	0.039*	
H50B	0.5700	0.5839	0.3239	0.039*	

### Atomic displacement parameters (Å^2^)

**Table:**

	*U*^11^	*U*^22^	*U*^33^	*U*^12^	*U*^13^	*U*^23^
Na1	0.0196 (7)	0.0196 (11)	0.0234 (7)	0.0009 (7)	0.0026 (5)	−0.0003 (7)
Na2	0.0203 (8)	0.0189 (10)	0.0223 (6)	−0.0018 (7)	0.0016 (6)	0.0016 (7)
N1	0.0154 (16)	0.0172 (12)	0.0182 (17)	0.0009 (15)	0.0026 (12)	0.0005 (15)
C2	0.015 (3)	0.0152 (18)	0.027 (3)	−0.0043 (15)	−0.002 (2)	−0.0050 (16)
C3	0.022 (3)	0.026 (2)	0.028 (3)	0.0014 (16)	0.005 (3)	−0.0027 (16)
C4	0.034 (2)	0.034 (2)	0.0177 (19)	−0.003 (2)	0.0063 (17)	−0.0016 (18)
C5	0.0275 (18)	0.0252 (17)	0.0256 (19)	−0.0014 (15)	−0.0027 (15)	0.0018 (15)
C6	0.0184 (16)	0.0214 (16)	0.0300 (19)	0.0002 (14)	−0.0039 (15)	−0.0012 (15)
O7	0.0259 (15)	0.0232 (11)	0.0173 (14)	0.0010 (14)	0.0057 (12)	0.0016 (14)
S8	0.0167 (4)	0.0223 (4)	0.0223 (4)	−0.0001 (3)	0.0008 (3)	−0.0001 (4)
S9	0.0176 (4)	0.0174 (4)	0.0334 (5)	0.0008 (3)	−0.0010 (4)	−0.0023 (4)
C10	0.020 (2)	0.0223 (17)	0.015 (2)	−0.0022 (17)	0.0002 (18)	0.0049 (17)
N11	0.0185 (15)	0.0199 (15)	0.0268 (17)	0.0000 (12)	−0.0002 (12)	−0.0030 (13)
C12	0.017 (2)	0.0306 (18)	0.030 (3)	0.003 (2)	−0.0022 (17)	−0.005 (2)
C13	0.025 (2)	0.029 (2)	0.020 (2)	−0.0078 (17)	−0.0006 (15)	−0.0038 (17)
C14	0.0280 (19)	0.0205 (17)	0.025 (2)	0.0015 (15)	0.0032 (15)	−0.0023 (14)
C15	0.0201 (17)	0.0221 (18)	0.0203 (18)	0.0025 (14)	−0.0007 (15)	0.0015 (14)
O16	0.0310 (17)	0.0227 (14)	0.0466 (18)	0.0028 (13)	0.0001 (15)	−0.0032 (13)
N17	0.017 (2)	0.0170 (13)	0.0162 (19)	0.0016 (14)	0.0036 (15)	0.0023 (15)
C18	0.0254 (17)	0.0142 (15)	0.0211 (18)	0.0072 (14)	−0.0027 (14)	0.0027 (13)
C19	0.036 (2)	0.0246 (17)	0.0183 (19)	0.0091 (16)	0.0015 (15)	0.0015 (14)
C20	0.040 (3)	0.034 (2)	0.023 (2)	0.012 (2)	0.0086 (18)	0.007 (2)
C21	0.025 (3)	0.028 (2)	0.035 (3)	0.0056 (17)	0.010 (2)	0.0114 (19)
C22	0.024 (2)	0.0194 (15)	0.026 (2)	−0.0023 (19)	0.0003 (17)	0.0041 (18)
O23	0.023 (2)	0.0225 (15)	0.020 (2)	−0.0003 (10)	0.0026 (16)	0.0006 (11)
S24	0.0188 (4)	0.0237 (4)	0.0321 (5)	0.0009 (3)	−0.0039 (4)	−0.0021 (4)
N25	0.022 (2)	0.0149 (13)	0.026 (2)	−0.0064 (14)	−0.0042 (16)	−0.0020 (15)
C26	0.014 (2)	0.0229 (16)	0.030 (2)	−0.0051 (19)	0.0006 (16)	−0.0032 (19)
C27	0.023 (2)	0.0294 (19)	0.036 (3)	0.001 (2)	0.004 (2)	−0.005 (2)
C28	0.039 (3)	0.039 (2)	0.025 (2)	−0.012 (2)	0.0154 (19)	−0.003 (2)
C29	0.044 (2)	0.0296 (18)	0.0219 (19)	−0.0083 (17)	−0.0064 (17)	0.0028 (16)
C30	0.0276 (18)	0.0170 (15)	0.0245 (19)	−0.0078 (14)	−0.0031 (15)	0.0011 (14)
O31	0.027 (2)	0.0212 (15)	0.0157 (19)	−0.0011 (10)	0.0056 (16)	0.0035 (10)
S32	0.0226 (5)	0.0258 (4)	0.0404 (5)	−0.0033 (4)	−0.0080 (4)	0.0061 (4)
C33	0.013 (2)	0.0204 (19)	0.018 (2)	−0.0027 (14)	0.0012 (18)	0.0048 (16)
N34	0.0184 (15)	0.0208 (15)	0.0177 (15)	−0.0004 (11)	0.0012 (12)	0.0011 (12)
C35	0.016 (2)	0.0321 (19)	0.029 (3)	0.006 (2)	0.0015 (17)	0.005 (2)
C36	0.028 (2)	0.025 (2)	0.034 (2)	0.0109 (17)	0.0112 (17)	0.0102 (17)
C37	0.0299 (19)	0.0197 (17)	0.031 (2)	0.0030 (16)	0.0082 (16)	0.0029 (15)
C38	0.0214 (18)	0.0237 (18)	0.0247 (19)	−0.0026 (14)	0.0021 (16)	0.0014 (15)
O39	0.0208 (14)	0.0211 (14)	0.0372 (16)	−0.0042 (11)	−0.0021 (13)	0.0015 (11)
S40	0.0160 (4)	0.0168 (4)	0.0338 (5)	−0.0014 (3)	−0.0001 (4)	0.0036 (4)
S41	0.0157 (4)	0.0201 (4)	0.0226 (4)	−0.0007 (3)	0.0008 (3)	0.0012 (4)
C42	0.017 (3)	0.0138 (18)	0.017 (3)	0.0040 (13)	0.002 (2)	−0.0006 (13)
N43	0.0181 (16)	0.0150 (12)	0.0211 (17)	0.0051 (15)	0.0019 (13)	−0.0011 (15)
C44	0.0158 (16)	0.0209 (16)	0.0291 (19)	−0.0009 (14)	0.0009 (14)	−0.0031 (14)
C45	0.0243 (18)	0.0305 (19)	0.026 (2)	0.0017 (15)	−0.0063 (15)	−0.0036 (16)
C46	0.034 (2)	0.0272 (18)	0.0185 (19)	0.004 (2)	0.0053 (16)	−0.0049 (17)
C47	0.019 (3)	0.024 (3)	0.021 (3)	0.0018 (12)	0.005 (2)	0.0008 (13)
O48	0.0208 (14)	0.0218 (10)	0.0183 (15)	−0.0017 (13)	0.0060 (11)	0.0025 (14)
O49	0.0188 (11)	0.0225 (12)	0.0399 (14)	0.0010 (10)	0.0010 (10)	0.0065 (11)
O50	0.0181 (11)	0.0247 (12)	0.0343 (13)	0.0037 (10)	−0.0003 (10)	−0.0038 (11)

### Geometric parameters (Å, °)

**Table:**

Na1—O31	2.328 (4)	C21—C22	1.366 (8)
Na1—O23	2.355 (4)	C21—H21	0.9500
Na1—O50	2.373 (3)	C22—H22	0.9500
Na1—O48^i^	2.459 (3)	N25—O31	1.321 (7)
Na1—O7	2.495 (3)	N25—C26	1.345 (6)
Na1—S32	2.990 (3)	N25—C30	1.387 (6)
Na1—H50B	2.6283	C26—C27	1.360 (7)
Na2—O23	2.332 (4)	C26—H26	0.9500
Na2—O31^ii^	2.335 (4)	C27—C28	1.397 (7)
Na2—O49	2.404 (3)	C27—H27	0.9500
Na2—O7	2.459 (3)	C28—C29	1.358 (6)
Na2—O48	2.473 (3)	C28—H28	0.9500
Na2—S24	2.999 (2)	C29—C30	1.409 (5)
N1—O7	1.316 (4)	C29—H29	0.9500
N1—C6	1.342 (4)	C30—S32	1.712 (4)
N1—C2	1.374 (7)	O31—Na2^i^	2.335 (4)
C2—C3	1.372 (10)	C33—N34	1.362 (6)
C2—S8	1.785 (7)	C33—C38	1.377 (4)
C3—C4	1.371 (8)	C33—S40	1.766 (5)
C3—H3	0.9500	N34—O39	1.317 (4)
C4—C5	1.382 (5)	N34—C35	1.353 (5)
C4—H4	0.9500	C35—C36	1.380 (6)
C5—C6	1.373 (4)	C35—H35	0.9500
C5—H5	0.9500	C36—C37	1.384 (5)
C6—H6	0.9500	C36—H36	0.9500
S8—S9	2.0536 (11)	C37—C38	1.389 (5)
S9—C10	1.758 (5)	C37—H37	0.9500
C10—N11	1.367 (6)	C38—H38	0.9500
C10—C15	1.383 (5)	S40—S41	2.0521 (11)
N11—O16	1.320 (4)	S41—C42	1.750 (7)
N11—C12	1.347 (5)	C42—N43	1.348 (7)
C12—C13	1.380 (6)	C42—C47	1.386 (10)
C12—H12	0.9500	N43—O48	1.332 (4)
C13—C14	1.379 (5)	N43—C44	1.358 (4)
C13—H13	0.9500	C44—C45	1.365 (4)
C14—C15	1.396 (5)	C44—H44	0.9500
C14—H14	0.9500	C45—C46	1.396 (5)
C15—H15	0.9500	C45—H45	0.9500
N17—O23	1.339 (7)	C46—C47	1.380 (8)
N17—C22	1.378 (6)	C46—H46	0.9500
N17—C18	1.378 (5)	C47—H47	0.9500
C18—C19	1.405 (4)	O48—Na1^ii^	2.459 (3)
C18—S24	1.716 (3)	O49—H49A	0.8987
C19—C20	1.375 (6)	O49—H49B	0.8936
C19—H19	0.9500	O50—H50A	0.9220
C20—C21	1.368 (7)	O50—H50B	0.8634
C20—H20	0.9500		
			
O31—Na1—O23	172.9 (2)	C12—C13—H13	120.3
O31—Na1—O50	94.98 (15)	C13—C14—C15	119.1 (3)
O23—Na1—O50	86.52 (14)	C13—C14—H14	120.5
O31—Na1—O48^i^	79.63 (13)	C15—C14—H14	120.5
O23—Na1—O48^i^	93.33 (13)	C10—C15—C14	120.0 (4)
O50—Na1—O48^i^	94.33 (12)	C10—C15—H15	120.0
O31—Na1—O7	106.45 (14)	C14—C15—H15	120.0
O23—Na1—O7	80.56 (13)	O23—N17—C22	116.8 (4)
O50—Na1—O7	87.02 (11)	O23—N17—C18	121.8 (4)
O48^i^—Na1—O7	173.66 (15)	C22—N17—C18	121.4 (4)
O31—Na1—S32	66.01 (13)	N17—C18—C19	116.5 (3)
O23—Na1—S32	114.50 (14)	N17—C18—S24	120.1 (3)
O50—Na1—S32	154.47 (11)	C19—C18—S24	123.4 (3)
O48^i^—Na1—S32	98.48 (10)	C20—C19—C18	122.2 (3)
O7—Na1—S32	82.68 (10)	C20—C19—H19	118.9
O31—Na1—Na2	148.47 (11)	C18—C19—H19	118.9
O23—Na1—Na2	38.50 (10)	C21—C20—C19	119.2 (4)
O50—Na1—Na2	86.09 (7)	C21—C20—H20	120.4
O48^i^—Na1—Na2	131.80 (9)	C19—C20—H20	120.4
O7—Na1—Na2	42.06 (9)	C22—C21—C20	120.0 (5)
S32—Na1—Na2	101.31 (5)	C22—C21—H21	120.0
O31—Na1—Na2^i^	37.85 (11)	C20—C21—H21	120.0
O23—Na1—Na2^i^	135.08 (11)	C21—C22—N17	120.7 (5)
O50—Na1—Na2^i^	96.66 (7)	C21—C22—H22	119.7
O48^i^—Na1—Na2^i^	41.79 (8)	N17—C22—H22	119.7
O7—Na1—Na2^i^	144.23 (9)	N17—O23—Na2	117.2 (2)
S32—Na1—Na2^i^	78.91 (4)	N17—O23—Na1	128.6 (3)
Na2—Na1—Na2^i^	173.05 (4)	Na2—O23—Na1	102.53 (19)
O31—Na1—H50B	113.5	C18—S24—Na2	90.25 (11)
O23—Na1—H50B	68.7	O31—N25—C26	117.9 (4)
O50—Na1—H50B	19.0	O31—N25—C30	120.2 (4)
O48^i^—Na1—H50B	101.5	C26—N25—C30	121.9 (5)
O7—Na1—H50B	77.9	N25—C26—C27	122.3 (5)
S32—Na1—H50B	159.6	N25—C26—H26	118.9
Na2—Na1—H50B	68.1	C27—C26—H26	118.9
Na2^i^—Na1—H50B	113.8	C26—C27—C28	118.5 (5)
O23—Na2—O31^ii^	173.9 (2)	C26—C27—H27	120.8
O23—Na2—O49	96.71 (16)	C28—C27—H27	120.8
O31^ii^—Na2—O49	86.74 (14)	C29—C28—C27	118.8 (4)
O23—Na2—O7	81.77 (13)	C29—C28—H28	120.6
O31^ii^—Na2—O7	93.04 (13)	C27—C28—H28	120.6
O49—Na2—O7	94.09 (12)	C28—C29—C30	123.2 (4)
O23—Na2—O48	105.84 (14)	C28—C29—H29	118.4
O31^ii^—Na2—O48	79.21 (13)	C30—C29—H29	118.4
O49—Na2—O48	87.67 (11)	N25—C30—C29	115.2 (3)
O7—Na2—O48	171.96 (15)	N25—C30—S32	120.9 (3)
O23—Na2—S24	66.19 (13)	C29—C30—S32	123.9 (3)
O31^ii^—Na2—S24	111.71 (14)	N25—O31—Na1	117.6 (2)
O49—Na2—S24	157.31 (10)	N25—O31—Na2^i^	127.9 (2)
O7—Na2—S24	97.82 (10)	Na1—O31—Na2^i^	104.43 (19)
O48—Na2—S24	83.23 (10)	C30—S32—Na1	90.28 (12)
O23—Na2—Na1	38.96 (11)	N34—C33—C38	120.2 (4)
O31^ii^—Na2—Na1	135.80 (10)	N34—C33—S40	111.6 (3)
O49—Na2—Na1	96.87 (8)	C38—C33—S40	128.2 (4)
O7—Na2—Na1	42.81 (8)	O39—N34—C35	121.9 (3)
O48—Na2—Na1	144.76 (9)	O39—N34—C33	117.5 (3)
S24—Na2—Na1	79.32 (4)	C35—N34—C33	120.6 (3)
O23—Na2—Na1^ii^	147.27 (11)	N34—C35—C36	120.0 (4)
O31^ii^—Na2—Na1^ii^	37.72 (10)	N34—C35—H35	120.0
O49—Na2—Na1^ii^	85.78 (7)	C36—C35—H35	120.0
O7—Na2—Na1^ii^	130.75 (9)	C35—C36—C37	120.7 (4)
O48—Na2—Na1^ii^	41.50 (8)	C35—C36—H36	119.6
S24—Na2—Na1^ii^	100.67 (5)	C37—C36—H36	119.6
Na1—Na2—Na1^ii^	173.05 (4)	C36—C37—C38	118.1 (4)
O7—N1—C6	122.1 (3)	C36—C37—H37	121.0
O7—N1—C2	117.8 (4)	C38—C37—H37	121.0
C6—N1—C2	120.1 (4)	C33—C38—C37	120.3 (4)
C3—C2—N1	121.2 (5)	C33—C38—H38	119.8
C3—C2—S8	130.7 (5)	C37—C38—H38	119.8
N1—C2—S8	108.0 (4)	C33—S40—S41	102.27 (16)
C4—C3—C2	118.4 (5)	C42—S41—S40	102.9 (2)
C4—C3—H3	120.8	N43—C42—C47	119.3 (5)
C2—C3—H3	120.8	N43—C42—S41	110.1 (4)
C3—C4—C5	120.2 (4)	C47—C42—S41	130.5 (5)
C3—C4—H4	119.9	O48—N43—C42	116.6 (4)
C5—C4—H4	119.9	O48—N43—C44	121.1 (3)
C6—C5—C4	120.0 (3)	C42—N43—C44	122.3 (4)
C6—C5—H5	120.0	N43—C44—C45	119.4 (3)
C4—C5—H5	120.0	N43—C44—H44	120.3
N1—C6—C5	120.1 (3)	C45—C44—H44	120.3
N1—C6—H6	119.9	C44—C45—C46	119.9 (3)
C5—C6—H6	119.9	C44—C45—H45	120.1
N1—O7—Na2	125.9 (2)	C46—C45—H45	120.1
N1—O7—Na1	123.6 (2)	C47—C46—C45	119.4 (4)
Na2—O7—Na1	95.13 (14)	C47—C46—H46	120.3
C2—S8—S9	101.8 (2)	C45—C46—H46	120.3
C10—S9—S8	101.62 (18)	C46—C47—C42	119.6 (5)
N11—C10—C15	119.3 (4)	C46—C47—H47	120.2
N11—C10—S9	111.9 (3)	C42—C47—H47	120.2
C15—C10—S9	128.7 (4)	N43—O48—Na1^ii^	123.8 (2)
O16—N11—C12	122.6 (3)	N43—O48—Na2	124.4 (2)
O16—N11—C10	116.7 (3)	Na1^ii^—O48—Na2	96.72 (14)
C12—N11—C10	120.7 (3)	Na2—O49—H49A	118.6
N11—C12—C13	121.1 (4)	Na2—O49—H49B	98.2
N11—C12—H12	119.5	H49A—O49—H49B	94.4
C13—C12—H12	119.5	Na1—O50—H50A	128.3
C14—C13—C12	119.4 (4)	Na1—O50—H50B	97.4
C14—C13—H13	120.3	H50A—O50—H50B	112.6
			
O31—Na1—Na2—O23	−177.5 (4)	S24—Na2—O23—N17	−43.5 (3)
O50—Na1—Na2—O23	89.3 (2)	Na1—Na2—O23—N17	−146.4 (5)
O48^i^—Na1—Na2—O23	−3.0 (2)	Na1^ii^—Na2—O23—N17	28.3 (5)
O7—Na1—Na2—O23	179.2 (3)	O49—Na2—O23—Na1	−92.64 (17)
S32—Na1—Na2—O23	−115.3 (2)	O7—Na2—O23—Na1	0.54 (18)
O31—Na1—Na2—O31^ii^	7.08 (14)	O48—Na2—O23—Na1	177.89 (15)
O23—Na1—Na2—O31^ii^	−175.4 (4)	S24—Na2—O23—Na1	102.91 (17)
O50—Na1—Na2—O31^ii^	−86.1 (2)	Na1^ii^—Na2—O23—Na1	174.73 (9)
O48^i^—Na1—Na2—O31^ii^	−178.4 (2)	O50—Na1—O23—N17	52.9 (4)
O7—Na1—Na2—O31^ii^	3.8 (2)	O48^i^—Na1—O23—N17	−41.2 (4)
S32—Na1—Na2—O31^ii^	69.24 (19)	O7—Na1—O23—N17	140.5 (4)
O31—Na1—Na2—O49	−85.3 (3)	S32—Na1—O23—N17	−142.1 (4)
O23—Na1—Na2—O49	92.2 (2)	Na2—Na1—O23—N17	141.0 (5)
O50—Na1—Na2—O49	−178.48 (13)	Na2^i^—Na1—O23—N17	−43.0 (5)
O48^i^—Na1—Na2—O49	89.20 (15)	O50—Na1—O23—Na2	−88.09 (17)
O7—Na1—Na2—O49	−88.60 (15)	O48^i^—Na1—O23—Na2	177.77 (17)
S32—Na1—Na2—O49	−23.15 (7)	O7—Na1—O23—Na2	−0.54 (18)
O31—Na1—Na2—O7	3.3 (3)	S32—Na1—O23—Na2	76.89 (19)
O23—Na1—Na2—O7	−179.2 (3)	Na2^i^—Na1—O23—Na2	175.96 (7)
O50—Na1—Na2—O7	−89.89 (15)	N17—C18—S24—Na2	−30.1 (3)
O48^i^—Na1—Na2—O7	177.8 (3)	C19—C18—S24—Na2	149.7 (3)
S32—Na1—Na2—O7	65.44 (13)	O23—Na2—S24—C18	33.22 (15)
O31—Na1—Na2—O48	179.0 (3)	O31^ii^—Na2—S24—C18	−152.94 (15)
O23—Na1—Na2—O48	−3.5 (2)	O49—Na2—S24—C18	−10.4 (3)
O50—Na1—Na2—O48	85.81 (18)	O7—Na2—S24—C18	110.60 (14)
O48^i^—Na1—Na2—O48	−6.51 (11)	O48—Na2—S24—C18	−77.44 (13)
O7—Na1—Na2—O48	175.7 (3)	Na1—Na2—S24—C18	71.81 (11)
S32—Na1—Na2—O48	−118.86 (18)	Na1^ii^—Na2—S24—C18	−115.26 (11)
O31—Na1—Na2—S24	117.3 (3)	O31—N25—C26—C27	178.8 (4)
O23—Na1—Na2—S24	−65.2 (2)	C30—N25—C26—C27	−0.9 (6)
O50—Na1—Na2—S24	24.16 (7)	N25—C26—C27—C28	1.4 (7)
O48^i^—Na1—Na2—S24	−68.16 (13)	C26—C27—C28—C29	0.1 (7)
O7—Na1—Na2—S24	114.04 (15)	C27—C28—C29—C30	−2.1 (6)
S32—Na1—Na2—S24	179.49 (8)	O31—N25—C30—C29	179.3 (3)
O7—N1—C2—C3	178.7 (4)	C26—N25—C30—C29	−0.9 (5)
C6—N1—C2—C3	−1.6 (6)	O31—N25—C30—S32	−2.2 (5)
O7—N1—C2—S8	−2.2 (4)	C26—N25—C30—S32	177.6 (3)
C6—N1—C2—S8	177.5 (3)	C28—C29—C30—N25	2.4 (5)
N1—C2—C3—C4	0.8 (6)	C28—C29—C30—S32	−176.0 (3)
S8—C2—C3—C4	−178.0 (3)	C26—N25—O31—Na1	−132.8 (3)
C2—C3—C4—C5	−0.1 (6)	C30—N25—O31—Na1	47.0 (5)
C3—C4—C5—C6	0.0 (6)	C26—N25—O31—Na2^i^	87.3 (5)
O7—N1—C6—C5	−178.8 (3)	C30—N25—O31—Na2^i^	−92.9 (4)
C2—N1—C6—C5	1.5 (5)	O50—Na1—O31—N25	117.2 (4)
C4—C5—C6—N1	−0.7 (5)	O48^i^—Na1—O31—N25	−149.3 (4)
C6—N1—O7—Na2	37.1 (5)	O7—Na1—O31—N25	28.9 (4)
C2—N1—O7—Na2	−143.2 (3)	S32—Na1—O31—N25	−45.1 (3)
C6—N1—O7—Na1	−90.7 (4)	Na2—Na1—O31—N25	26.6 (5)
C2—N1—O7—Na1	89.0 (4)	Na2^i^—Na1—O31—N25	−148.3 (5)
O23—Na2—O7—N1	−139.2 (3)	O50—Na1—O31—Na2^i^	−94.44 (18)
O31^ii^—Na2—O7—N1	44.0 (3)	O48^i^—Na1—O31—Na2^i^	−0.94 (18)
O49—Na2—O7—N1	−43.0 (3)	O7—Na1—O31—Na2^i^	177.20 (15)
S24—Na2—O7—N1	156.4 (3)	S32—Na1—O31—Na2^i^	103.27 (18)
Na1—Na2—O7—N1	−138.7 (3)	Na2—Na1—O31—Na2^i^	174.91 (10)
Na1^ii^—Na2—O7—N1	45.0 (3)	N25—C30—S32—Na1	−28.0 (3)
O23—Na2—O7—Na1	−0.50 (16)	C29—C30—S32—Na1	150.4 (3)
O31^ii^—Na2—O7—Na1	−177.35 (15)	O31—Na1—S32—C30	32.34 (16)
O49—Na2—O7—Na1	95.70 (12)	O23—Na1—S32—C30	−155.44 (16)
S24—Na2—O7—Na1	−64.94 (12)	O50—Na1—S32—C30	−12.3 (2)
Na1^ii^—Na2—O7—Na1	−176.35 (6)	O48^i^—Na1—S32—C30	106.95 (14)
O31—Na1—O7—N1	−38.2 (3)	O7—Na1—S32—C30	−79.35 (13)
O23—Na1—O7—N1	140.5 (3)	Na2—Na1—S32—C30	−117.25 (11)
O50—Na1—O7—N1	−132.5 (3)	Na2^i^—Na1—S32—C30	69.83 (11)
S32—Na1—O7—N1	24.1 (3)	C38—C33—N34—O39	175.1 (4)
Na2—Na1—O7—N1	140.0 (3)	S40—C33—N34—O39	−5.8 (5)
Na2^i^—Na1—O7—N1	−35.2 (4)	C38—C33—N34—C35	−3.7 (7)
O31—Na1—O7—Na2	−178.21 (15)	S40—C33—N34—C35	175.5 (3)
O23—Na1—O7—Na2	0.50 (16)	O39—N34—C35—C36	−175.5 (4)
O50—Na1—O7—Na2	87.46 (13)	C33—N34—C35—C36	3.2 (6)
S32—Na1—O7—Na2	−115.94 (10)	N34—C35—C36—C37	−0.6 (6)
Na2^i^—Na1—O7—Na2	−175.27 (8)	C35—C36—C37—C38	−1.6 (6)
C3—C2—S8—S9	4.4 (4)	N34—C33—C38—C37	1.4 (7)
N1—C2—S8—S9	−174.6 (2)	S40—C33—C38—C37	−177.6 (4)
C2—S8—S9—C10	−91.6 (2)	C36—C37—C38—C33	1.1 (6)
S8—S9—C10—N11	−159.6 (3)	N34—C33—S40—S41	−155.9 (3)
S8—S9—C10—C15	24.0 (5)	C38—C33—S40—S41	23.2 (5)
C15—C10—N11—O16	173.5 (4)	C33—S40—S41—C42	−89.8 (2)
S9—C10—N11—O16	−3.3 (5)	S40—S41—C42—N43	−173.9 (2)
C15—C10—N11—C12	−7.2 (7)	S40—S41—C42—C47	5.9 (4)
S9—C10—N11—C12	176.0 (3)	C47—C42—N43—O48	177.9 (3)
O16—N11—C12—C13	−176.1 (4)	S41—C42—N43—O48	−2.2 (4)
C10—N11—C12—C13	4.7 (7)	C47—C42—N43—C44	−3.2 (5)
N11—C12—C13—C14	0.4 (6)	S41—C42—N43—C44	176.7 (3)
C12—C13—C14—C15	−2.7 (5)	O48—N43—C44—C45	−179.3 (3)
N11—C10—C15—C14	4.9 (7)	C42—N43—C44—C45	1.8 (5)
S9—C10—C15—C14	−178.9 (4)	N43—C44—C45—C46	0.7 (5)
C13—C14—C15—C10	0.0 (5)	C44—C45—C46—C47	−1.8 (5)
O23—N17—C18—C19	−178.6 (3)	C45—C46—C47—C42	0.4 (6)
C22—N17—C18—C19	−1.6 (5)	N43—C42—C47—C46	2.0 (5)
O23—N17—C18—S24	1.3 (5)	S41—C42—C47—C46	−177.9 (3)
C22—N17—C18—S24	178.3 (3)	C42—N43—O48—Na1^ii^	−141.7 (3)
N17—C18—C19—C20	1.6 (5)	C44—N43—O48—Na1^ii^	39.4 (4)
S24—C18—C19—C20	−178.2 (3)	C42—N43—O48—Na2	89.4 (4)
C18—C19—C20—C21	−1.4 (6)	C44—N43—O48—Na2	−89.6 (4)
C19—C20—C21—C22	1.0 (6)	O23—Na2—O48—N43	−38.0 (3)
C20—C21—C22—N17	−1.0 (6)	O31^ii^—Na2—O48—N43	138.5 (3)
O23—N17—C22—C21	178.5 (4)	O49—Na2—O48—N43	−134.3 (3)
C18—N17—C22—C21	1.4 (6)	S24—Na2—O48—N43	24.8 (3)
C22—N17—O23—Na2	−133.1 (3)	Na1—Na2—O48—N43	−35.7 (4)
C18—N17—O23—Na2	44.0 (5)	Na1^ii^—Na2—O48—N43	139.4 (3)
C22—N17—O23—Na1	90.6 (5)	O23—Na2—O48—Na1^ii^	−177.42 (15)
C18—N17—O23—Na1	−92.3 (4)	O31^ii^—Na2—O48—Na1^ii^	−0.86 (16)
O49—Na2—O23—N17	120.9 (3)	O49—Na2—O48—Na1^ii^	86.26 (13)
O7—Na2—O23—N17	−145.9 (4)	S24—Na2—O48—Na1^ii^	−114.56 (11)
O48—Na2—O23—N17	31.4 (4)	Na1—Na2—O48—Na1^ii^	−175.12 (8)

Symmetry codes: (i) *x*, *y*+1, *z*; (ii) *x*, *y*−1, *z*.

### Hydrogen-bond geometry (Å, °)

**Table:**

Cg3 is the centroid of the N34/C33–C35 ring.

**Table:**

*D*—H···*A*	*D*—H	H···*A*	*D*···*A*	*D*—H···*A*
O49—H49A···O16^iii^	0.90	1.91	2.784 (3)	165
O49—H49A···N11^iii^	0.90	2.67	3.417 (3)	141
O49—H49B···S32^ii^	0.89	2.37	3.198 (2)	154
O50—H50A···O39^iv^	0.92	1.88	2.798 (3)	177
O50—H50A···N34^iv^	0.92	2.62	3.455 (3)	151
O50—H50B···S24	0.86	2.33	3.193 (2)	174
C29—H29···Cg1^v^	0.95	2.89	3.618 (4)	134

Symmetry codes: (iii) *x*+1/2, −*y*+1, *z*; (ii) *x*, *y*−1, *z*; (iv) *x*−1/2, −*y*+1, *z*; (v) −*x*+3/2, *y*+1, *z*+1/2.
